# Impaired Expression of Tetraspanin 32 (TSPAN32) in Memory T Cells of Patients with Multiple Sclerosis

**DOI:** 10.3390/brainsci10010052

**Published:** 2020-01-17

**Authors:** Maria Sofia Basile, Emanuela Mazzon, Katia Mangano, Manuela Pennisi, Maria Cristina Petralia, Salvo Danilo Lombardo, Ferdinando Nicoletti, Paolo Fagone, Eugenio Cavalli

**Affiliations:** 1 Department of Biomedical and Biotechnological Sciences, University of Catania, Via S. Sofia 89, 95123 Catania, Italy; sofiabasile@hotmail.it (M.S.B.); kmangano@unict.it (K.M.); manuela.pennisi@unict.it (M.P.); salvo.lombardo.sdl@gmail.com (S.D.L.); ferdinic@unict.it (F.N.); 2 IRCCS Centro Neurolesi “Bonino-Pulejo”, Via Provinciale Palermo, Contrada Casazza, 98124 Messina, Italy; emanuela.mazzon@irccsme.it (E.M.); m.cristinapetralia@gmail.com (M.C.P.); eugenio.cavalli@irccsme.it (E.C.)

**Keywords:** TSPAN32, tetraspanins, multiple sclerosis, cellular immunity, memory T cells

## Abstract

Tetraspanins are a conserved family of proteins involved in a number of biological processes. We have previously shown that Tetraspanin-32 (TSPAN32) is significantly downregulated upon activation of T helper cells via anti-CD3/CD28 stimulation. On the other hand, TSPAN32 is marginally modulated in activated Treg cells. A role for TSPAN32 in controlling the development of autoimmune responses is consistent with our observation that encephalitogenic T cells from myelin oligodendrocyte glycoprotein (MOG)-induced experimental autoimmune encephalomyelitis (EAE) mice exhibit significantly lower levels of TSPAN32 as compared to naïve T cells. In the present study, by making use of ex vivo and in silico analysis, we aimed to better characterize the pathophysiological and diagnostic/prognostic role of TSPAN32 in T cell immunity and in multiple sclerosis (MS). We first show that TSPAN32 is significantly downregulated in memory T cells as compared to naïve T cells, and that it is further diminished upon ex vivo restimulation. Accordingly, following antigenic stimulation, myelin-specific memory T cells from MS patients showed significantly lower expression of TSPAN32 as compared to memory T cells from healthy donors (HD). The expression levels of TSPAN32 was significantly downregulated in peripheral blood mononuclear cells (PBMCs) from drug-naïve MS patients as compared to HD, irrespective of the disease state. Finally, when comparing patients undergoing early relapses in comparison to patients with longer stable disease, moderate but significantly lower levels of TSPAN32 expression were observed in PBMCs from the former group. Our data suggest a role for TSPAN32 in the immune responses underlying the pathophysiology of MS and represent a proof-of-concept for additional studies aiming at dissecting the eventual contribution of TSPAN32 in other autoimmune diseases and its possible use of TSPAN32 as a diagnostic factor and therapeutic target.

## 1. Introduction

Tetraspanins are a conserved family of proteins involved in several biological processes, such as the regulation of cellular adhesion, motility, cancer metastasis, signal transduction, and activation [1,2]. Tetraspanins comprise four transmembrane (TM) domains. TM domains 1 and 2 flank a small extracellular loop (SEL), while TM3 and TM4 flank a large extracellular loop (LEL). TM domains are typically involved in the interaction with non-tetraspanin molecules. The juxtamembrane cysteine residues in the cytoplasmic domains contribute to the formation of tetraspanin-enriched microdomains (TEMs), while the cytoplasmic regions provide links to cytoskeletal and signaling molecules [3]. Several immune-related proteins take part in TEMs, including pattern recognition receptors, co-stimulatory molecules, Major Histocompatibility Complex molecules and T cell receptor-associated proteins (reviewed in [2]). The tetraspanins Cluster of Differentiation 82 (CD82), CD9, CD63, CD81, and CD53 exert a co-stimulatory role in T cells [4,5], whereas cells deficient for CD37, CD151, and CD81 have been shown to be hyperproliferative following stimulation [6,7,8]. Tarrant and colleagues [9] have shown that T cells from Tssc6 Tetraspanin-32 (TSPAN32)-deficient mice have increased responses upon stimulation, and have proposed that TSPAN32 may negatively regulate peripheral T-lymphocyte activation. Along the same lines, we have previously shown that TSPAN32 expression is significantly reduced upon cell activation, although in Treg cells, TSPAN32 levels undergo minor changes. Moreover, significantly lower levels of TSPAN32 were found in encephalitogenic T cells from myelin oligodendrocyte glycoprotein (MOG)-Induced experimental autoimmune encephalomyelitis (EAE) mice. Finally, ex vivo-activated circulating CD4 T cells from MS patients showed lower levels of TSPAN32 as compared to cells from healthy donors [10]. 

Multiple sclerosis (MS) is the most frequent immuno-inflammatory disorder of the central nervous system, characterized by immune cell infiltration, microglia activation and progressive demyelination, with consequent neurological deficits. It is well-known that increased conversion from naïve to memory cells can be observed in MS [11] and that most of the myelin-reactive T cells are present in the memory T cell subset [12]. It has been also shown that memory T cells are activated independently of CD28 co-stimulation [13,14]. In the present paper, we aimed to better characterize the pathophysiological role of TSPAN32 in cellular immunity and in MS. To this aim, by making use of ex vivo and in silico analysis, we have evaluated the expression levels of TSPAN32 in memory T cells from healthy donors and MS patients, both in inactive state and upon activation. Next, we determined the diagnostic and prognostic value of TSPAN32 in the peripheral blood mononuclear cells (PBMCs) of MS patients. Our analysis demonstrates that TSPAN32 is significantly downregulated in memory T cells as compared to naïve T cells, and that it is further diminished upon ex vivo restimulation. In addition, following antigenic stimulation, myelin-specific memory T cells from MS patients exhibited significantly lower expression of TSPAN32 as compared to memory T cells from healthy donors (HD). Further, the expression levels of TSPAN32 was significantly downregulated in PBMCs from drug-naïve MS patients as compared to HD, irrespective of the disease state. Finally, we observed a moderate but significantly reduced expression of TSPAN32 in PBMCs from MS patients undergoing early relapses in comparison to those from patients with a longer course of stable disease. 

## 2. Materials and Methods

### 2.1. Ex Vivo Study 

#### 2.1.1. Cell Isolation and Real-Time PCR

Mononuclear cells were obtained from the peripheral blood of healthy donors (HD) (*n* = 7) by step-gradient centrifugation, using the Ficoll−Hypaque medium (Sigma Aldrich, Milano, Italy), as per manufacturer’s instructions. CD4 + CD45RA + CD45RO − CD25 + CD127^low^ cells (naive Treg cells), CD4 + CD45RA − CD45RO + CD25 + CD127^low^ cells (memory Treg cells), CD4 + CD45RA − CD45RO + CD25 − CD127 + cells (memory Teff cells), and CD4 + CD45RA + CD45RO − CD25 − CD127 + cells (naive Teff cells) were enriched by magnetic beads sorting, obtaining a cell purity of at least 95%. In another set of experiments, memory Teff cells from 3 healthy donors were activated by plate-bound anti-CD3 (10 μg/mL) and anti-CD28 (5 μg/mL) for 12 h.

#### 2.1.2. Real-Time PCR

Total RNA was extracted and gene expression levels were determined by real-time PCR. 2 μg of total RNA were reverse-transcribed with a High-Capacity cDNA Reverse Transcription Kit (Applied Biosystems, Monza, Italy) in a 20 μL reaction volume, and real-time PCR was performed using the SYBR Green PCR Master Mix (Applied Biosystems, Monza, Italy), 200 nM forward and 200 nM reverse primers, and 20 μg cDNA. Relative gene expression levels were obtained using the formula: 2^−ΔΔ*C*t^, where ΔΔ*C*t = (*C*t_target gene_ − *C*t_beta-actin_) stimulated cells – (*C*t_target gene_ − *C*t_beta-actin_) control cells.

### 2.2. In Silico Analysis

**Figure 1:**
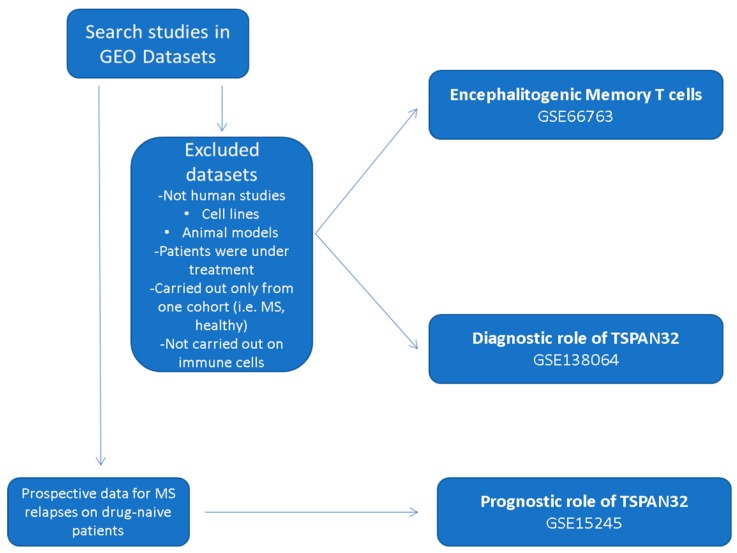
Flowchart of the in silico study.

The Gene Expression Omnibus (GEO; https://www.ncbi.nlm.nih.gov/gds) browser was interrogated using the MeSH (Medical Subject Headings) term “Multiple Sclerosis”. Datasets were manually excluded if the studies were not performed on human subjects, if the patients enrolled were under immunosuppressive/immunomodulatory treatment, and if the cell types analyzed were not immune cells. For the evaluation of the expression levels of TSPAN32 in encephalitogenic memory T cells, and the evaluation of the diagnostic role of TSPAN32, datasets carried out only on one cohort of subjects (i.e., MS patients and healthy donors) were excluded. For the afore-mentioned reasons, the analysis was then carried out on the GSE66763 and the GSE138064, respectively. For the determination of the prognostic properties of TSPAN32 in predicting MS relapses, the GSE15245 was selected as it is the only dataset including prospective data on disease evolution. A flowchart of the in silico study design is provided as Figure 1. The characteristics of the datasets used are described in the following sections. 

#### 2.2.1. TSPAN32 in Memory T Cells from MS Patients

The GSE66763 dataset was used to investigate the expression levels of TSPAN32 in circulating memory T cells from MS patients [15]. The dataset included whole-genome RNA sequencing data of C-C Motif Chemokine Receptor 6 (CCR6)^+^ memory (CD45RA − CD45RO + CD25 − CCR6+) CD4+ T from 3 Human Leukocyte Antigen – DR isotype (HLA-DR)4+ healthy subjects and 5 HLA-DR4+ MS patients. Cells were amplified by PhytoHaemAgglutinin (PHA) and Interleukin (IL)-2 and stimulated by irradiated autologous monocytes and DR4 myelin peptides Myelin Oligodendrocyte Glycoprotein ((MOG)_97–109_ and ProteoLipid Protein (PLP)_180–199_). Patients were immunotherapeutic naïve or had not received treatment for at least 12 months. Cell proliferation was determined and the highest proliferated wells were chosen for DR4 tetramers staining (MOG_97–109_-tetramers and PLP_180–199_-tetramers). Then, myelin tetramer+ and tetramer− cells were sorted and lysed for the extraction of RNA and subsequent RNA sequencing. Gene expression is shown as log_2_ Fragments Per Kilobase of transcript per Million mapped (FPKM) values.

#### 2.2.2. TSPAN32 in PBMCs from MS Patients

In order to investigate the expression levels of TSPAN32 in PBMCs from MS patients in both stable and active disease, as compared to healthy donors, we interrogated the GSE138064 dataset [16]. The dataset included transcriptomic data from therapy-naïve Relapse-Remitting (RR) MS patients (10 with stable MS, age 45.2  ±  2.6, 8/2 female/male, and 9 during relapse, age 46.3  ±  3.5, 8/2 female/male). Eight healthy controls were included, age 42.3 ±  4.8, 5/3 female/male.

#### 2.2.3. Predictive Analysis of TSPAN32 in MS

In order to evaluate the relationship between expression levels of TSPAN32 and the time to relapse in MS patients, we interrogated the GSE15245 dataset that included whole-genome transcriptomic profiles of PMBCs from 51 drug-naïve MS patients [17]. The patient’s age was 38.5 ± 1.4, with a mean Expanded Disability Status Scale (EDSS) score of 2.4 ± 0.2. The Affymetrix Human Genome U133A 2.0 Array was used for the generation of the dataset and raw data were preprocessed using the robust multi-array average (RMA) algorithm. Sample population was sorted based on the expression levels of TSPAN32 and log-rank test was applied to evaluate differences in the percentage of patients developing acute relapses in a 1500-day time frame.

### 2.3. Statistical Analysis

Data are shown as mean ± SD and statistical analysis was performed using either a Student’s *t*-test or one-way ANOVA followed by Fisher’s Least Significant Difference test. Correlation analysis was performed using the non-parametric Spearman’s test. Hierarchical clustering was used to determine the relative distance of samples using Pearson's correlation as similarity comparison. The self organizing map (SOM) algorithm was used for the unsupervised identification of clusters of commonly modulated genes [18]. Distance metric for SOM was Pearson’s correlation, with random genes initialization, Gaussian neighborhood, and 2000 iterations. The linear model for microarray (LIMMA) algorithm was used to evaluate statistical significance for differences in RNA sequencing data [19]. As the experimental design and the information provided are different for the three whole-genome transcriptomic datasets here analyzed, and in consideration that no additional datasets with overlapping experimental layouts are currently available in publicly available databases, a meta-analysis cannot be performed. Gene ontology and gene term enrichment analysis was conducted using the web-based utility, Metascape [20]. GraphPad Prism 8 and MeV (version 4.9) software programs were used for the statistical analysis and the generation of the graphs.

## 3. Results

### 3.1. TSPAN32 in Memory T Cells 

**Figure 2:**
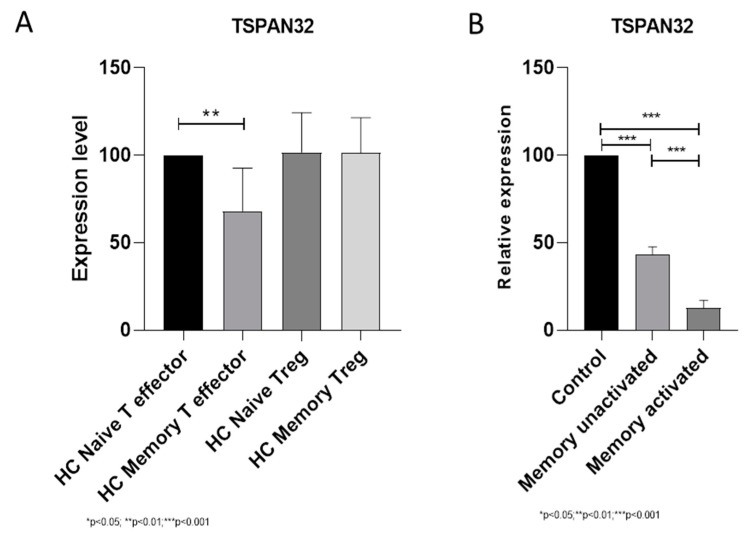
(**A**) Basal expression levels of Tetraspanin-32 (TSPAN32) in naïve T effector, Treg, memory T effector, and memory Treg cells from healthy donors; (**B**) modulation of TSPAN32 expression in memory T cells upon reactivation. ** *p* < 0.01, *** *p* < 0.001.

When analyzing the expression levels of TSPAN32 in memory CD4+ T cells from healthy donors, we observed significantly lower levels of TSPAN32 in memory T effector cells as compared to naïve T cells (*p* < 0.01) (Figure 2A). On the other hand, no modulation was observed in memory Treg cells (Figure 2A). We also wanted to determine whether a modulation of TSPAN32 could be found upon restimulation. As shown in Figure 2B, restimulation of memory T cells is associated to a significant down regulation in TSPAN32 levels (*p* < 0.001) (Figure 2B). Similar data have been obtained from the analysis of the GSE22886 dataset (Table S1).

**Figure 3:**
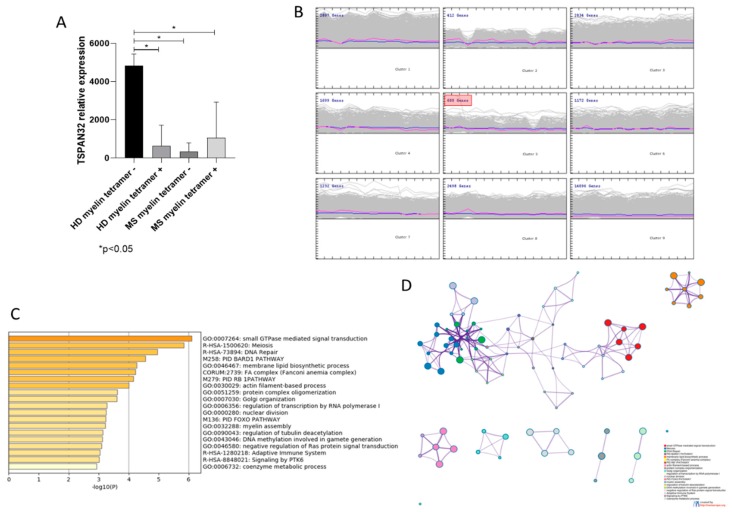
(**A**) TSPAN32 expression in memory T cells from healthy donors and multiple sclerosis (MS) patients; * *p* < 0.05; (**B**) clusters of genes obtained from the self organizing map (SOM) analysis; (**C**) most enriched biological processes by genes commonly regulated with TSPAN32, as obtained from SOM analysis; (**D**) network showing the interconnection among the most enriched biological processes by genes commonly regulated with TSPAN32, obtained from SOM analysis.

Next, we wanted to determine the expression of TSPAN32 in memory CD4+ T cells from HLA-DR4+ MS patients, following amplification by PHA and IL-2 and stimulation by irradiated autologous monocytes and DR4 myelin peptides. As shown in Figure 3A, significant lower levels of TSPAN32 were observed in tetramer+ memory T cells from MS patients as compared to tetramer- memory T cells from HD (*p* < 0.05). Similarly, comparable levels of TSPAN32 were observed in tetramer+ memory T cells from HD (Figure 3A). SOM analysis identified 599 genes that clustered together with TSPAN32 (Cluster 5) (Figure 3B). Gene ontology revealed that the most significant enriched terms were “Small GTPase-mediated signal transduction”, “Meiosis”, “DNA repair”, “BARD1 pathway” and “Membrane lipid biosynthetic process” (Figure 3B–D). Interestingly, significantly lower TSPAN32 levels were also observed in tetramer- memory T cells from MS patients (Figure 3A). As LIMMA analysis revealed significant transcriptomic differences between tetramer- MS memory T cells and tetramer- HD memory T cells, with enrichment of several immune-related biological processes (Figure S1A,B), and HCL analysis clustered together tetramer- and tetramer+ memory T cells from MS patients (Figure S1C), the reduced TSPAN32 levels may be associated to a reduced activation threshold of memory T cells from MS patients, and could explain the underlying autoimmune process.

### 3.2. TSPAN32 Expression in PBMCs from MS Patients 

**Figure 4:**
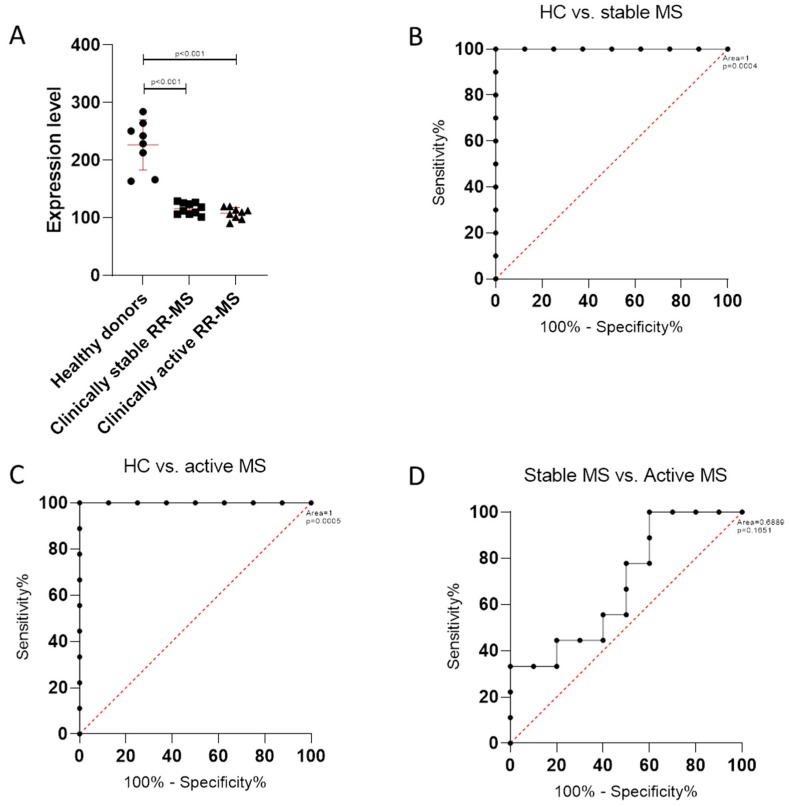
(**A**) TSPAN32 in peripheral blood mononuclear cells (PBMCs) from healthy donors and RRMS patients in stable and relapsing disease; (**B**) receiver operating characteristic (ROC) curve for TSPAN32 in healthy controls (HC) and multiple sclerosis patients in stable disease; (**C**) receiver operating characteristic (ROC) curve for TSPAN32 in healthy controls (HC) and multiple sclerosis patients in active disease; (**D**) receiver operating characteristic (ROC) curve for TSPAN32 in multiple sclerosis patients in stable and active disease.

In order to evaluate whether a modulation in TSPAN32 levels could be observed in peripheral immune cells from MS patients, we interrogated the GSE138064 dataset. As shown in Figure 4A, a significant reduction in TSPAN32 expression was observed in PBMCs from MS patients in both stable and relapsing disease (*p* < 0.001) (Figure 4A). Receiver operating characteristic (ROC) analysis confirmed the diagnostic ability of TSPAN32 to discriminate MS from HD, entailing a *p* < 0.001 (Figure 4B,C). No significant differences were instead observed when comparing TSPAN32 levels in PBMCs from patients in stable disease as compared to PBMCs from patients in exacerbation (Figure 4A). Accordingly, ROC curve area was 0.6889, entailing a *p* = 0.1651 (Figure 4D). This is in accordance with data from the GSE19224 dataset, that show an adjusted *p* value > 0.99 and a log_2_(fold) change of 0.276 for TSPAN32 expression levels when comparing PBMCs from MS patients in stable versus relapsing disease (https://www.ncbi.nlm.nih.gov/geo/geo2r/?acc=GSE19224).

**Figure 5:**
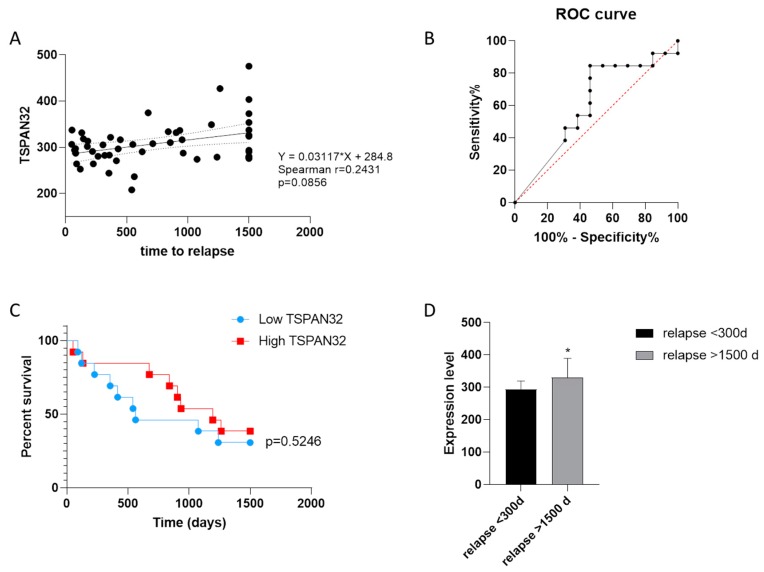
(**A**) Correlation between TSPAN32 expression in PBMCs from drug-naïve MS patients and the time-to-relapse; Spearman correlation value (r) and p value (p) are indicated. (**B**) receiver operating characteristic (ROC) curve for the evaluation of the prognostic value of TSPAN32 in predicting relapses in MS patients; (**C**) log-rank analysis for time-to-relapse in patients expressing low and high levels of TSPAN32 in PBMCs, respectively; (**D**) expression levels of TSPAN32 in MS patients undergoing early exacerbation of the disease (<300 days) or with longer stable disease (relapse > 1500 days).

Finally, we evaluated whether the different transcriptional levels of TSPAN32 in PBMCs from MS patients could promote disease exacerbation or protect MS patients from acute relapses. Non-parametric correlation between TSPAN32 and the time-to-relapse revealed a trend of direct correlation, which did not reach the statistical significance (*p* = 0.0856) (Figure 5A). ROC curve area was 0.6036, entailing a *p* = 0.3695 (Figure 5B). However, Log-rank analysis performed on patients divided into two groups based on the expression level of TSPAN32 in PBMCs (referred as High and Low TSPAN32) showed that a trend of protection from acute relapses was observed in patients expressing higher TSPAN32 levels (Figure 5C). In addition, significantly lower levels of TSPAN32 were found in PBMCs from MS patients developing exacerbation of the disease before 300 days as compared with patients who underwent relapses later than 1500 days (Figure 5D).

## 4. Discussion

Diverse members of the TSPAN family have been shown to be involved in the regulation of both the innate and adaptive immune responses. For instance, CD81 is involved in the formation of the immune synapse, providing a link between the Antigen Presenting Cells and the T cells [21,22], while CD37 and CD151 promote antigen presentation and regulate the costimulatory signaling pathways [23].

In a TSPAN32 (Tssc6)-deficient mouse model, despite normal hemopoiesis, T cell proliferation and responses are significantly augmented [9]. It has also been observed that the activity of T cells from mice double knockout for CD37 and TSPAN32 are upregulated, and that the dendritic cell stimulation capacity is increased as compared to single knockout, suggesting a cooperative role for these two tetraspanins in controlling T cell-mediated immunity [24]. These findings suggest that TSPAN32 might contribute to shape cellular immunity. In our previous work, we have described that T cells express a baseline level of TSPAN32, favoring the maintenance of an inactive state, which is decreased following CD3-mediated signaling [10].

By means of in silico and ex vivo analyses, in this study we wanted to gain further insights into the role of TSPAN in the biology and physiology of memory T cells and evaluated whether its expression was altered in memory T cells from MS patients as compared to HD. We also studied the possible diagnostic and prognostic value of TSPAN32 expression in PBMC of MS patients, on the course of the disease. The use of whole-genome expression databases has been largely exploited [25,26,27,28] for the characterization of pathogenic pathways and to identify therapeutic targets for a variety of disorders, including immunoinflammatory and autoimmune diseases [29,30,31,32,33,34,35,36], cancer [37,38,39], and has allowed dismantling pathogenetic pathways [40,41,42], along with the identification of novel tailored therapeutic targets [43,44,45,46]. 

MS is an autoimmune/immunoinflammatory disorder sustained by activated, myelin-specific T cells that migrate into the central nervous system (CNS), promoting inflammation. The characterization of the phenotype of myelin-specific immune cells is, therefore, crucial for the elucidation of MS pathogenesis [47,48,49].

In the present study, we have first analyzed the expression levels of TSPAN32 in circulating memory T cells from HD and we show that significantly lower levels of TSPAN32 can be observed in memory T effector cells but not in memory Treg cells. This is in line with our previous observation that only a marginal downregulation of TSPAN32 occurs in Treg cells, upon activation [10]. Interestingly, following the in vitro reactivation of memory T effector cells, TSPAN32 expression levels further decreased. The differential pattern of expression and modulation of TSPAN32 in Treg cells has yet to be deciphered.

Next, we analyzed the expression of TSPAN32 in autoreactive T cells from MS patients. As shown by Cao et al., myelin-reactive T cells from MS patients are prevalently from the memory CCR6+ T cell population, and are characterized by the secretion of larger amounts of proinflammatory cytokines as compared to T cells from HD. As expected, the expression levels of TSPAN32 in myelin-reactive MS tetramer-positive T cells resulted significantly lower than those in tetramer-negative memory T cells from HD. On the other hand, a significant lower expression of TSPAN32 was found in MS tetramer-negative memory T cells, comparable to that of MS tetramer-positive T cells. Although this may be counterintuitive, the observation that the transcriptomic features of the MS tetramer-negative memory T cells are more closely related to those of the MS tetramer-positive memory T cells than those of HD tetramer-negative memory T cells suggests that a lower activation threshold characterizes memory T cells from MS patients. Notably, a similar trend of reduced TSPAN32 levels was also observed in myelin-reactive HD tetramer-positive T cells. It is already known that MS patients and healthy subjects share a similar number of circulating myelin-reactive T cells. The lower levels of TSPAN32 in these cells suggest that regardless of the activation state of the T cells, the engagement of the TCR with the cognate ligand is sufficient to modulate the expression of TSPAN32. This is in accordance with our previous data, showing that the CD3-mediated signaling is sufficient to downregulate TSPAN32 gene expression.

Finally, we found diminished TSPAN32 levels in PBMCs from MS patients, both in stable and active disease, as compared to HD. No differences were however found between patients in stable versus relapsing disease. In addition, only a moderate reduction in the time-to-relapse was observed in patients expressing higher levels of TSPAN32 when MS patients were divided into two groups on the basis of their level of TSPAN32 expression in PBMC (referred as High and Low TSPAN32). 

However, it was possible to observe that those with high expression had a moderate but significant protection from acute relapses. In agreement with this observation, significantly lower levels of TSPAN32 were found in PBMCs from MS patients developing exacerbation of the disease before 300 days, as compared with patients who underwent relapses later than 1500 days. Overall, our data suggest that the defective expression of TSPAN32 may characterize different T cell subsets of MS patients, including memory T cells, and that this may contribute to trigger anti-myelin immune responses. Along with our previous publication [10], this new transcriptomic analysis strengthens our hypothesis that defective TSPAN32 expression may represent an additional important immunopathogenetic abnormality that may play a role in the pathogenesis of at least some cases of MS. 

It should also be pointed out that, although all of the in silico data have been generated from third-party reanalysis of whole-genome transcriptomic datasets previously validated by the respective original authors, the number of biological replicates in each of these datasets is relatively low. Therefore, although statistical significance has been achieved in most cases of our analyses, the data warrant to be confirmed from a larger population of MS patients. Along this line of research, it will be interesting to study if and how the current disease modifying therapies influence the course of the disease by modulating TSPAN32 expression. In a similar manner, the expression profile of TSPAN32 in secondary progressive and primary progressive MS seems of interest. Additional studies may also be warranted to dismantle whether defective expression of TSPAN32 is also observed in other T cell-mediated autoimmune diseases.

Moreover, further effort is required to understand the molecular pathways involved in the regulation of the immune responses exerted by TSPAN32. Up to now, no drugs targeting tetraspanins have received approval for the use in the clinical setting, but many strategies have been explored, including the use of monoclonal antibodies, recombinant soluble large extracellular loops or RNA interference (RNAi) (reviewed in [50]). Therefore, it is reasonable that several chances for tailored-specific intervention will be available in the future. Additionally, a deeper understanding of the mechanisms that control TSPAN32 expression could be pursued for their possible efficacy in patients suffering from MS.

## 5. Conclusions

Our data suggest a role for TSPAN32 in the immune responses underlying the pathophysiology of MS and represent a proof-of-concept for additional studies aiming at dissecting the eventual contribution of TSPAN32 in other autoimmune diseases and its possible use of TSPAN32 as a diagnostic factor and therapeutic target.

## Supplementary Materials

The following are available online at https://www.mdpi.com/2076-3425/10/1/52/s1, Figure S1: Comparative transcriptomic profile of MS tetramer- memory T cells; Table S1: Significant genes in the GSE22886 dataset.
