# Supplementary material for: Impaired Expression of Tetraspanin 32 (TSPAN32) in Memory T Cells of Patients with Multiple Sclerosis
Source: Brain Sci. 2020 Jan 17;10(1):52. doi: 10.3390/brainsci10010052 (PMC7016636; doi:10.3390/brainsci10010052)
Supplement: Supplementary file 1 [file brainsci-10-00052-s001.zip › brainsci-668862-supplementary/Supplementary Figure Legend.docx]

Supplementary Figure Legend

**FigureS1. Comparative transcriptomic profile of MS tetramer negative memory T cells.** A) Most enriched Biological Processes by genes differentially regulated between MS tetramer negative and HD tetramer negative memory T cells; B) Network showing the interconnection among the most enriched Biological Processes enriched by genes differentially regulated between MS tetramer negative and HD tetramer negative memory T cells; C) Hierarchical Clustering Analysis of the transcriptomic profile of memory T cells in MS patients and healthy controls
