# Supplementary figures and images for: Impaired Expression of Tetraspanin 32 (TSPAN32) in Memory T Cells of Patients with Multiple Sclerosis
Source: Brain Sci. 2020 Jan 17;10(1):52. doi: 10.3390/brainsci10010052 (PMC7016636; doi:10.3390/brainsci10010052)

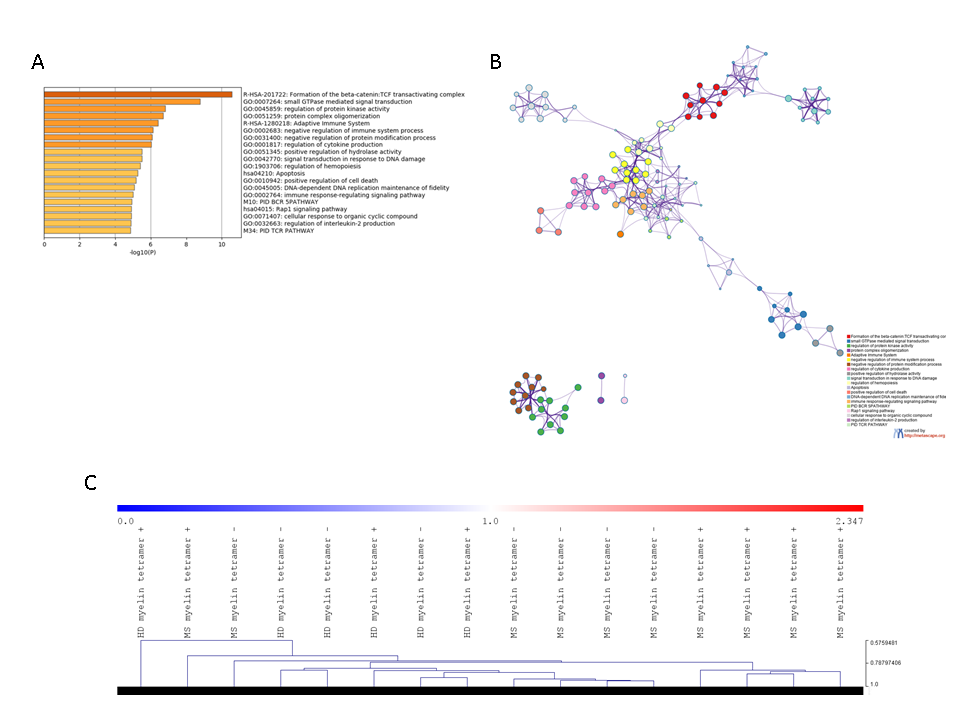

Supplement: Supplementary file 1 [file brainsci-10-00052-s001.zip › brainsci-668862-supplementary/Suppl Fig 1 TSPAN32 MS memory.tif]
